# Histopathology Study of Patients with Delayed Mustard Gas Keratopathy 30 Years After Exposure

**DOI:** 10.30699/IJP.2022.538950.2722

**Published:** 2022-08-13

**Authors:** Seyed-Hashem Daryabari, Hossein Aghamollaei, Fahimeh Asadi Amoli, Khosrow Jadidi, Hamid Tebyanian

**Affiliations:** 1 *Chemical Injuries Research Center, Systems biology and Poisonings Institute, Baqiyatallah University of Medical Sciences, Tehran, Iran*; 2 *Eye Research Center, Farabi Eye Hospital, Tehran University of Medical Sciences, Tehran, Iran*; 3 *Vision Health Research Center, Semnan University of Medical Sciences, Semnan, Iran*; 4 *Research Center for Prevention of Oral and Dental Diseases, Baqiyatallah University of Medical Sciences, Tehran, Iran*

**Keywords:** Mustard Gas, Cornea, Keratopathy, Fibrosis

## Abstract

**Background & Objective:**

Delayed mustard gas keratopathy (DMGK) is the main chronic outcome in eye-chemical injured patients. The aim of this study was the histopathological evaluation of mustard-exposed cornea after more than 30 years.

**Methods:**

Fourteen corneas after Lamellar keratoplasty were evaluated in this study. Corneal tissues were prepared by histologic methods and stained by H&E.

**Results:**

The main histopathological findings in these cases were the presence of severe stromal edema and corneal scar. In the sections with visible superficial epithelium, subepithelial bullae formation was observed. Focal or diffuse disruption of Bowman's membrane and replacement with fibrosis were also seen. There was no evidence of stromal vascularization and inflammation in all specimens.

**Conclusion:**

After more than 30 years, an extensive corneal scar is seen in sulfur mustard exposed patients. Scar tissue without vascularization and fibroblastic proliferation is the main finding in the sulfur mustard exposed cornea. This pathology result is different from other scars. No evidence of inflammation or immune cell infiltration should be considered in managing DMGK.

## Introduction

Sulfur Mustard is a chemical agent with alkylating and blistering effects that was widely used for the first time in World War I by Germans and then again by Iraqi forces in the Iran-Iraq war, affecting the lives of thousands of civilians (1). Mustard gas is easily absorbed through the skin because of its fat solubility, reacts with the aqueous environment of cells, and produces hydrochloric acid. Moreover, it alkylates intracellular proteins and enzymes and damages DNA, disrupting cell function and ultimately leading to cell death (2). Other known altered mechanisms include oxidative stress and inflammation pathways (3-5). According to the General Director of the Chemical Injuries of the Martyrs and Victims Affairs Foundation of Iran, about 63,000 approved chemical veterans are currently struggling with the effects of this gas in Iran. Although damages to the respiratory system are the main cause of mortality in mustard gas injured patients, the eyes are the most sensitive organ to mustard gas and can be affected with concentrations ten times less than those required to cause respiratory or skin damage (6, 7). In a study by Balali-Mood *et al.*, 65% of chemical veterans still suffered from ophthalmic complications even 30 years after the initial exposure to sulfur mustard (8).

Ocular exposure to mustard gas can follow three distinct paths. Most patients experience acute complications, including eye pain, itching, redness, foreign body sensation, burning, anterior uveitis, conjunctivitis, photophobia, and temporary blindness (9-11). These symptoms usually resolve after a few weeks without any sequela. However, some patients continue to have ocular symptoms for several years that may cause neovascularization, corneal haze, scarring, corneal dystrophy, limbal stem cells defect, corneal thinning, and limbus ischemia, and lipid and amyloid deposition in the cornea. The third group of patients develops delayed symptoms. In other words, they experience an asymptomatic period of even several years after the acute phase of intoxication, and symptoms similar to those of chronic keratitis begin to appear (12, 13). The chronic and delayed-type reaction constitutes a spectrum of ocular problems called mustard gas keratopathy (MGK). Currently, no definitive treatment exists for ocular complications of sulfur mustard. Treatments for MGK include pharmacological and surgical therapies such as artificial tears, anti-inflammatory drugs, immunomodulatory drugs, human blood derivatives, amniotic membrane transplantation, tarsorrhaphy, stem cell transplantation, and corneal transplantation (14, 15).

The pathologic effects of sulfur mustard on the respiratory system have been extensively studied; however, only a handful of pathology studies of the ocular complications exist (16-18). Understanding the changes that occur in the tissues after exposure to sulfur mustard can lead to a better understanding of the disease and help us in developing curative treatments for these patients. The most recent histopathological study of MGK was ten years ago by Kanavi et al., which reported the involvement of all layers of cornea from endothelium to epithelium, but more severe involvement of anterior layers (17). Hence, here we report pathological findings in a case series of patients with MGK that underwent lamellar keratoplasty thirty years after the initial exposure.

## Material and Methods

Fourteen formalin-fixed corneal buttons from patients with chronic and delayed MGK were obtained from the ophthalmology department thirty years after the exposure. All the patients had sufficient evidence of mustard gas-related keratopathy on slit-lamp examinations, including limbal ischemia, limbal vascular telangiectasia, corneal scarring, stromal thinning, and intrastromal deposits. Furthermore, they had definite documents for exposure to mustard gas during the Iran-Iraq war in the Veterans Foundation. Informed consent was obtained from all participants.

Initially, the tissues were processed and embedded in paraffin wax. Thin sections of 4 µm thickness were prepared and stained with Hematoxylin & Eosin (H&E) (19), periodic acid Schiff, Congo red, and positive Trichrome Masson. A single ophthalmic pathologist (F.A A) examined the stained slides using light microscopy (Olympus BX43, Tokyo, Japan)**.**

## Results

Fourteen corneal buttons from 14 patients, who underwent lamellar keratoplasty due to delayed mustard gas keratopathy (DMGK), were evaluated. The mean age at the time of lamellar keratoplasty was 55.92±8.08 years, and the mean interval between transplantation surgery and mustard gas exposure was 32.73 ±0.88 years. 

Almost all of the specimens were composed of the Bowman's layer and superficial stroma of cornea without deep stroma, Descemet's membrane, and endothelium. Superficial epithelium in some specimens was not seen or showed atrophy.

**Fig. 1:**
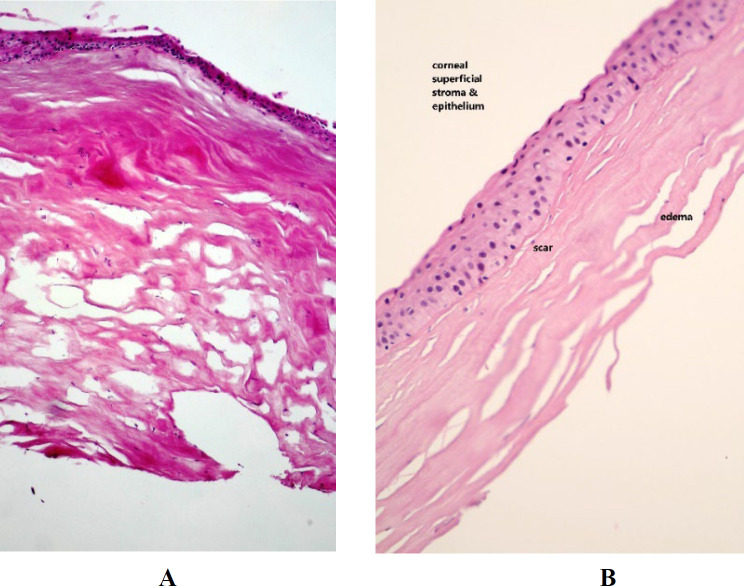
1A: Corneal tissue with a scar, edema, Bowman's layer disruption, H&E staining (× 400), 1B: Stromal scar and edema was marked

**Fig. 2:**
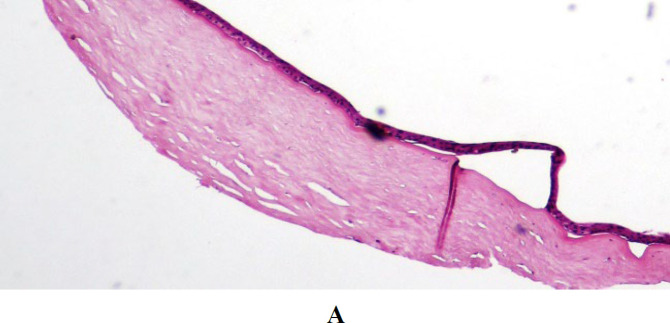
Corneal tissue with a scar, subepithelial bulla formation, 2A: H&E staining (×100), 2B: (×400). 2C: Epithelial edema with subepithelial bulla

The main histopathological findings in all of the specimens were the presence of severe stromal edema and corneal scar (Figure 1). In the sections with visible superficial epithelium, subepithelial bullae formation (Bullous keratopathy) was seen (Figure 2A,B). Focal or diffuse disruption of Bowman's membrane (3A) and replacement with fibrosis was also observed (3B).

There was no evidence of stromal vascularization or inflammation in any specimens. In one specimen, epithelium showed hyperplasia with scattered dyskeratotic cells without any neoplastic or dysplastic changes (3C)

Deposition of amorphous eosinophilic materials in the superficial epithelium was seen in one specimen after negative PAS, Congo-Red, and positive trichrome Masson staining, which was consistent with fibrosis and scar tissue (Figure 4).

**Fig. 3:**
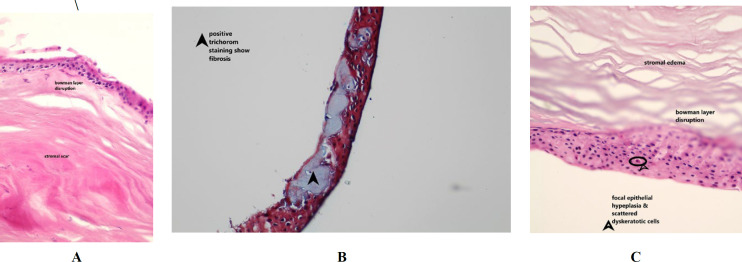
Corneal tissue with scar, edema, Bowman's layer disruption (3A) corneal subepithelial fibrosis (3B) confirmed by trichrome staining( ×400). Superfacial epithelium shows hyperplasia and scattered dyskeratotic cells (3C), H&E staining (×400). The dyskeratotic cell is marked by the arrow

**Fig. 4.**
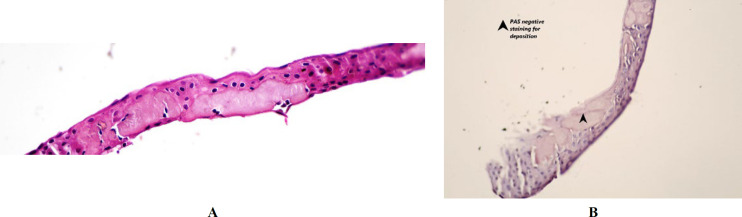
Deposition of amorphous eosinophilic materials in the DMGK cornea. 4A: H&E staining (×400) 4B: PAS negative staining (×400).

## Discussion

In this study, we assessed the histopathologic changes that occurred in the anterior corneal layers of patients with delayed effects of mustard gas approximately thirty years after the initial exposure. All of our patients had developed DMGK and required lamellar keratopathy to restore their vision. The predominant finding in all of the reviewed specimens was the presence of severe stromal edema and corneal scar. Moreover, fibrosis of Bowman's layer and bullous keratopathy was also observed in most of our specimens. An important finding was the absence of stromal vascularization and inflammation in all of our samples. Dysplastic changes were not found in any of our patients' specimens.

Mustard gas causes damage to the cornea initially due to its direct alkylating properties and the proteases and chemical mediators released by the damaged cell (20). Defect in iron-dependent regulations, pathological changes in vascular endothelial growth factor (VEGF) expression, reduced the concentration of antioxidant proteins, and apoptosis activation was reported as the possible reasons for DMGK (21). This damage is exacerbated by secondary conditions that develop in these patients, such as dry eye disease and limbal stem cell deficiency (22). Some studies showed the role of inflammation in delayed corneal damage caused by mustard gas (17, 23, 24). However, in our study, none of our specimens demonstrated evidence of inflammation or immune cell infiltration. Although the previous study showed chronic inflammation in histological evaluation of corneal samples of veterans with DMGK, they stated that damages of DMGK cannot be explained by inflammation and the alkylating effects of the gas are the main culprit (16). Their study's main feature of corneal epithelium was corneal conjunctival epithelium progress and thinning of the cornea. However, inflammation and vascularity of corneal stroma were evident in around half of their specimens. Considering that our specimens lacked deep stromal layers, this difference can be justified. Moreover, our study evaluated the histopathology of patients almost 30 years after initial exposure, whereas the previous study evaluated patients around 15 years after the exposure; therefore, increased time from exposure may be responsible for the lack of signs of inflammation in our study. This may point toward the fact that as time passed from the initial exposure, the immune system could control the inflammation. This may be an important factor for choosing the appropriate treatments and avoiding corticosteroids in patients with an older exposure time.

Initially, it was thought that mustard gas only damages the anterior surface of the cornea and does not involve the deeper layers; however, the involvement of posterior layers of the cornea in mustard gas intoxication has been shown. Javadi *et al.* demonstrated endothelial cell loss in patients with MGDK that underwent penetrating keratoplasty (PKP) (23). Moreover, Kanavi et al. showed mild to severe endothelial cell loss in 75% of patients that underwent PKP due to MGDK (17). This loss of endothelial cells causes dysfunction of the corneal endothelial barrier, which may contribute to corneal edema and the inability of the cornea to regenerate stroma (25, 26). However, we collected specimens from patients that underwent lamellar keratoplasty. Thus, we could not investigate deeper layers of stroma, Descemet's membrane, and endothelium in our patients. Our study had some limitations; firstly, we did not use immunohistochemical studies to evaluate our samples further. Moreover, our samples did not include deeper layers of cornea and endothelium; therefore, our results are limited only to the epithelium and superficial layers of the stroma. 

## Conclusion

We reported the histopathological changes of DMGK thirty years after the initial exposure. Our results showed severe stromal edema and corneal scarring without signs of inflammation or infiltration of immune cells suggesting that the detrimental effects of mustard gas in DMGK are mainly attributable to the alkylating effects of the mustard gas itself, and subsequent inflammation is not the main cause of corneal injury.

## Conflict of Interest

The authors declared no conflicts of interest.

## Funding

The authors did not receive support from any organization for the submitted work.

## Ethics Approval

Informed consent was obtained from all individual participants included in the study. All procedures performed in studies involving human participants were in accordance with the ethical standards of the institutional and/or national research committee and with the 1964 Helsinki Declaration and its later amendments or comparable ethical standards.
